# The Usage of ANN for Regression Analysis in Visible Light Positioning Systems

**DOI:** 10.3390/s22082879

**Published:** 2022-04-08

**Authors:** Neha Chaudhary, Othman Isam Younus, Luis Nero Alves, Zabih Ghassemlooy, Stanislav Zvanovec

**Affiliations:** 1 Instituto de Telecomunicações and Departamento de Electrónica, Telecomunicações e Informática, Universidade de Aveiro, 3810-193 Aveiro, Portugal; nero@ua.pt; 2 Optical Communications Research Group, Faculty of Engineering and Environment, Northumbria University, Newcastle upon Tyne NE1 8ST, UK; othman.younus@northumbria.ac.uk (O.I.Y.); z.ghassemlooy@northumbria.ac.uk (Z.G.); 3 Department of Electromagnetic Field, Faculty of Electrical Engineering, Czech Technical University in Prague, 16627 Prague, Czech Republic; xzvanove@fel.cvut.cz

**Keywords:** visible light communication (VLC), visible light positioning, multipath reflections, non-linear least square, artificial neural network (ANN), Bayesian regularization

## Abstract

In this paper, we study the design aspects of an indoor visible light positioning (VLP) system that uses an artificial neural network (ANN) for positioning estimation by considering a multipath channel. Previous results usually rely on the simplistic line of sight model with limited validity. The study considers the influence of noise as a performance indicator for the comparison between different design approaches. Three different ANN algorithms are considered, including Levenberg–Marquardt, Bayesian regularization, and scaled conjugate gradient algorithms, to minimize the positioning error ($\varepsilon_{p}$) in the VLP system. The ANN design is optimized based on the number of neurons in the hidden layers, the number of training epochs, and the size of the training set. It is shown that, the ANN with Bayesian regularization outperforms the traditional received signal strength (RSS) technique using the non-linear least square estimation for all values of signal to noise ratio (SNR). Furthermore, in the inner region, which includes the area of the receiving plane within the transmitters, the positioning accuracy is improved by 43, 55, and 50% for the SNR of 10, 20, and 30 dB, respectively. In the outer region, which is the remaining area within the room, the positioning accuracy is improved by 57, 32, and 6% for the SNR of 10, 20, and 30 dB, respectively. Moreover, we also analyze the impact of different training dataset sizes in ANN, and we show that it is possible to achieve a minimum $\varepsilon_{p}$ of 2 cm for 30 dB of SNR using a random selection scheme. Finally, it is observed that $\varepsilon_{p}$ is low even for lower values of SNR, i.e., $\varepsilon_{p}$ values are 2, 11, and 44 cm for the SNR of 30, 20, and 10 dB, respectively.

## 1. Introduction

The necessity for indoor location-based services has been growing over the past decades due to its significance in the development of various applications, such as smart home appliances, robots, supermarkets, shopping malls, hospitals, etc. Various conventional positioning techniques are based on radio frequency (RF) technologies; for instance, the global positioning system has been used in outdoor environments. However, in indoor environments, it suffers from multipath-induced fading, which can affect the accuracy of the position estimation significantly [1,2]. A number of RF-based positioning systems have also been introduced including Bluetooth [3], ultrasound [4], wireless local area network [5], ultra-wide band [5], and RF identification [6].

Light-emitting diodes (LEDs)-based visible light communication (VLC) systems have been introduced in recent years, which have shown great potential in achieving high-precision indoor positioning due to the use of optical signals. These systems are known as visible light positioning (VLP), which allows the usage of pre-installed LED luminaries as transmitters (Txs) in indoor environments [7]. VLP systems are considered as an emerging and cost-effective solution compared with other technologies. VLP also leverages the use of well-developed algorithms, which have been developed for other technologies [8], including the angle of arrival (AOA), time of arrival (TOA), proximity, scene analysis, and received signal strength (RSS) [9]. RSS, AOA, and TOA have been explored in VLP systems with the $\varepsilon_{p}$ of 10 to 40 cm, where $\varepsilon_{p}$ represents the positioning error, which is the difference between the actual and estimated output [10]. RSS-based positioning systems are much simpler for implementation compared with TOA and AOA-based positioning systems due to the fact that, they do not need highly accurate transceiver synchronization or a receiver (Rx) with efficient detection of the incidence angle [11]. Therefore, most of the previous studies have been focused on RSS-based VLP systems [12,13,14,15], where the strength of the received power is used to estimate the Rx’s position. Numerous research works have reported $\varepsilon_{p}$ close to 1 cm in the past three years [15,16,17]. The relatively simpler algorithms, such as proximity and scene analysis, trade simplicity with accuracy and are most appropriate for low accuracy systems.

Different estimation approaches have been used to estimate the Rx’s position. For instance, in [18], two conventional methods relying on linear least squares (LLS) and non-linear least squares (NLLS) were used for the position estimation. However, NLLS and LLS achieved the $\varepsilon_{p-\min}$ values of 46.42 and 55.89 cm, respectively, where $\varepsilon_{p-\min}$ represents the minimum positioning error achieved. An efficient RSS-based VLP algorithm was proposed in [16] to estimate the three- dimensional location of an Rx, combining two-dimensional trilateration with the NLLS. The computational time for NLLS is limited to approximately 17 ms, which is further reduced to less than 2 ms using a fast search algorithm.

Recently, an artificial neural network (ANN) has been utilized in RSS-based positioning systems. In [17], both RSS and ANN methods were proposed to achieve an accurate indoor VLP system with a diffuse optical channel. An accuracy of 6.4 cm was achieved with the averaged $\varepsilon_{p}$ being ~13 times smaller than RSS-based positioning system. In addition, a low-cost indoor VLP system was proposed using a machine learning algorithm in [6], which achieved an $\varepsilon_{p}$ of 3.7 cm with a height tolerance of 15 cm in line of sight (LoS) environment. In [19], a new 2-D ANN-based VLP system was proposed, where the LEDs were grouped into blocks, and the block coordinates were encoded using under-sampled modulation. A camera was used as an Rx to decode the block coordinate, and the system achieved a mean $\varepsilon_{p}$ of 1.5 cm in LoS channel. In [20], a VLP system based on the RSS and a deep ANN-based Bayesian regularization VLP system was proposed, where only the LoS transmission was considered. The results showed that, using only 20 training points a minimum $\varepsilon_{p}$ of 3.4 cm was achieved. In [21], an ANN-based approach was proposed exploiting the distortions caused by inaccurate modeling (i.e., phase and intensity models) in both phase difference of arrival and RSS-based positioning systems. The pre-trained models were applied to the ANN-based VLP system for reduced complexity and enhanced robustness, showing an $\varepsilon_{p}$ of 12 cm in an indoor LoS channel.

However, in many previous works, the effects of noise and multipath were not fully and consistently considered. For example, the works reported in [20,21] considered only LoS paths in the analysis of positioning performance without taking into account the multipath nature of the channel. Note, for systems using Txs and Rxs with a wide beam and a field of view (FOV), respectively, the impact of multipath reflections is inevitable and therefore must be considered as was reported in [18]. The results showed that, $\varepsilon_{p}$ values of 0.4 and 46.4 cm were achieved for the entire room without and with multipath reflections, respectively. Moreover, the impact of noise was investigated in [22], but the considered signal-to-noise ratio (SNR) was very high (i.e., 30 dB). Alternatively, in [23] the non-line of sight (NLoS) was considered under a very low power noise level (i.e., −140 to −180 dBm), where the minimum $\varepsilon_{p}$ of 0.05 cm was achieved by analytically solving the Lambertian transmission equation group. In [24], both multipath reflections and the impact of noise were considered, where$\;\varepsilon_{p}$ of 28 cm was achieved, although at a high SNR of 30 dB. Therefore, the impact of multipath reflections should be considered as it severely reduces the accuracy of the VLP system. Although the influence of NLoS on the system performance has been studied extensively and reported in the literature [25,26], not much has been done on the power distance relation, which is more complex. For regression analysis and position estimation, several machine learning approaches can be used.

The aim of this work is to investigate the utilization of ANN for regression analysis in the VLP system. A comprehensive study is conducted about the optimization of an ANN for VLP systems and a complete assessment of its performance. The error performance of the proposed system is evaluated by considering the noise over a wide range of SNR. For that, three different ANN algorithms, including Levenberg–Marquardt, Bayesian regularization, and scaled conjugate gradient, are explored to minimize the $\varepsilon_{p}$ of the proposed VLP system. The error performance is analyzed and compared with the traditional RSS technique, which uses an NLLS algorithm along with a polynomial regression model [26]. Firstly, the proposed ANN is optimized based on the number of neurons in the hidden layers (HLs) and the number of training epochs. Finally, we analyze the noise performance of the proposed system in comparison with the traditional approaches. We show that, the ANN with Bayesian regularization outperforms the traditional RSS technique using NLLS for a wide range of SNR. Moreover, we also analyze the impact of different training dataset sizes when training the neural network. We also observed an improvement in the positioning accuracy for the inner region by 43, 55, and 50% compared to 57, 32, and 6% in the outer region for the SNR values of 10, 20, and 30 dB, respectively.

The main contribution of this work is the performance evaluation and the design process of already existing ANN algorithms in the VLP systems considering a multipath channel, which has not been reported previously. In addition, we have optimized the proposed ANN model based on different parameters, such as the number of neurons in the hidden layers, the number of training epochs, and the size of the training set, which is proven to improve the positioning accuracy of the VLP system.

The rest of the paper is organized as follows; Section 2 presents the system model, the positioning algorithms, and the polynomial regression approach in detail. The ANN used for position estimation and different training algorithms are presented in Section 3. In Section 4, simulation results are discussed in detail, and finally, Section 5 concludes the paper.

## 2. VLP System Modelling

### 2.1. System Model

**Figure 1:**
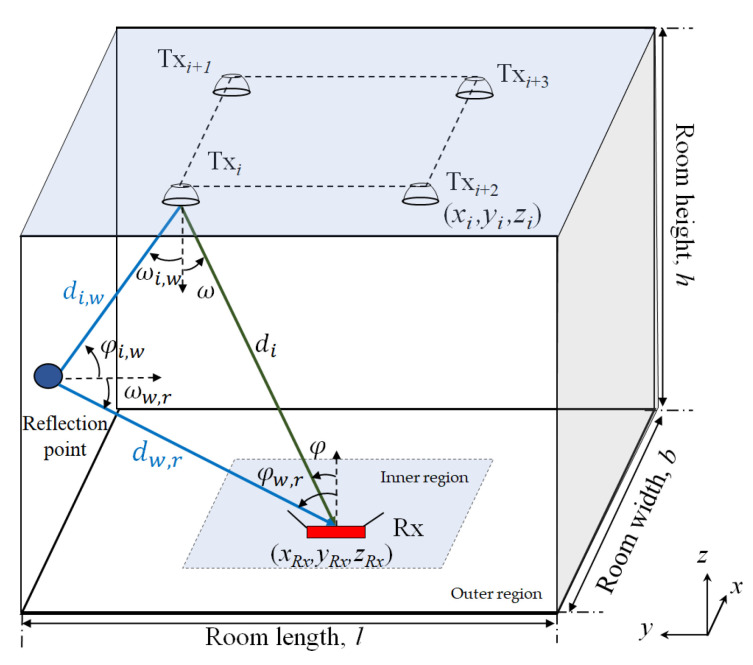
A VLP system with system configuration.

The proposed system consists of a standard empty room with several LED-based Txs and a single photodiode (PD)-based Rx, which is facing upwards, as depicted in Figure 1. The Txs and Rxs are placed on the ceiling and floor levels at heights, *h_t_* and *h_r_* of three and zero meters, respectively, from the ground. In the channel, we consider signals from both LoS and NLoS transmission paths. Note, for the NLoS, we have limited the reflections to the first order for: (i) the sake of simplicity [27]; and (ii) to contain most of the transmitted power [28]. In this work, we have adopted a simple Lambertian model with a *v* of 1 [29].

**Figure 2:**
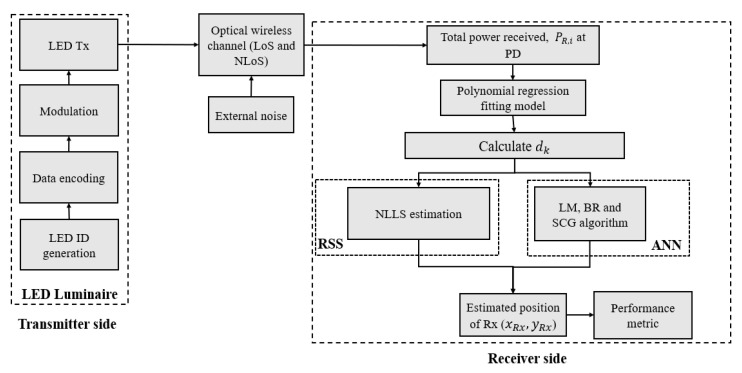
Block diagram of the proposed system.

The block diagram of the proposed scheme is depicted in Figure 2. We have not considered the synchronization issue and have assumed that each Tx transmits a unique ID information, which is encoded and modulated in the on-off keying (OOK) signal format, and at the Rx the received power $P_{R,i}$ due to each Tx being determined using correlation methods, which are given by [26]:

(1) $$P_{R,i}=\sum P_{\mathrm{LoS},i}+\sum P_{\mathrm{NLoS},i}+n_{G},\;$$

where $P_{\mathrm{LoS},i}$ and $P_{\mathrm{NLoS},i}$ are the received power from the *i*^th^ Tx due to LoS and NLoS paths, respectively, and $n_{G}$ is the additive white Gaussian noise power with a zero mean and variance $\sigma^{2}$ i.e., *N*(0,$\;\sigma^{2}$), which arise from the thermal noise, and dark current, signal, and background radiation-induced shot noises. Note, in VLC systems, the latter is the dominant noise source.

**Figure 3:**
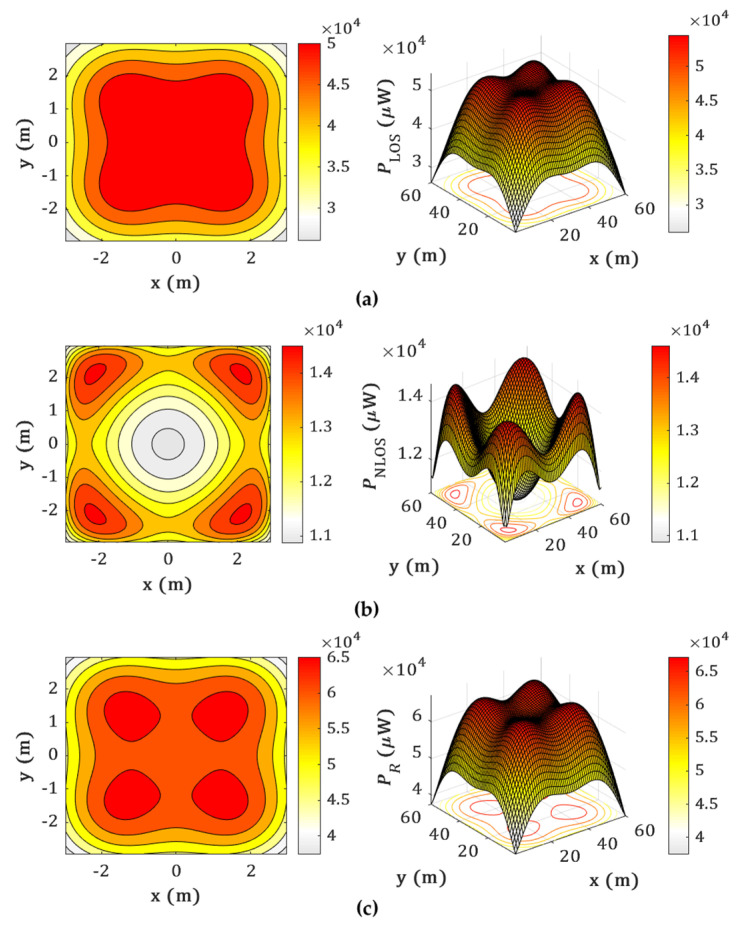
The received power distributions for the proposed system for: (**a**) LoS; (**b**) NLoS; and (**c**) LoS and NLoS links.

Figure 3 depicts the received power distribution for LoS, NLoS, and LoS with NLoS transmission paths. As illustrated in Figure 3a, for the LoS, the power is the highest directly beneath the Txs. The power decreases gradually with the user moving toward the corners and walls of the room. Figure 3b shows that, for the NLoS paths, power distributions are the highest along the walls, thus resulting in a slight rise in the total power received at the Rx near the walls. Figure 3c depicts the total power at Rx from both LoS and NLoS paths showing higher peak and average power level compared to Figure 3a,b. Note that, the received power from the NLoS paths leads to the overestimation of the transmission distances and, therefore, further degrades the positioning accuracy in the localization process. 

The received power from LoS path can be expressed as [30]:

(2) $$\sum P_{\mathrm{LoS},i}=\overset{I}{\underset{i=1}{\sum }}\frac{m+1}{2\pi }RA_{r}P_{t,i}\frac{\cos^{m}\left(\omega_{i}\right)\cos\left(\varphi \right)}{{∥d_{i}∥}^{2}}T_{s}\left(\varphi \right)g\left(\varphi \right),$$

where $d_{i}$ is the distance between the *i*^th^ Tx and the Rx, $\omega_{i}$ is the irradiance angle from the *i*^th^ Tx to the Rx, *φ*, and R are the incident angle and PD responsivity, respectively. $P_{t,i}$ is the transmit power from the *i*^th^ Tx and $A_{r}$ is the area of the PD. *T_s_*(φ) and *g*(φ) are the transmittance function and the concentrator gain of the Rx, respectively, that are considered to be unity for simplicity’s sake. Lambertian order is given by:

(3) $$v=-\frac{\ln\left(2\right)}{\ln\left(\cos\left(\mathrm{HPA}\right)\right)},$$

where HPA refers to the half-power angle for the light source. The RSS algorithm incorporates a distance estimation step based on the total received power $P_{R,i}$, where the distance between the *i*^th^ Tx and the Rx is estimated as:

(4) $$d_{i}=\sqrt{r_{i}^{2}+h^{2}},$$

where $r_{i}$ is the horizontal distance from the *i*^th^ Tx to the Rx and *h* is the difference in height between the Tx and Rx, i.e., (*h_t_* − *h_r_*). The received power from the first order reflection is given by [31]:

(5) $$\sum P_{\mathrm{NLoS},i}=\overset{I}{\underset{i=1}{\sum }}\underset{\mathrm{wall}}{\sum }\begin{array}{c} \left(\frac{m+1}{2\pi^{2}}\right)\rho RA_{r}P_{t,i}A_{\mathrm{ref}}\frac{\cos^{m}\left(\omega_{i,w}\right)\cos\left(\varphi_{i,w}\right)}{∥d_{i,w}{∥}^{2}∥d_{w,r}{∥}^{2}}\\ \times \cos\left(\omega_{w,r}\right)\cos\left(\varphi_{w,r}\right)T_{s}\left(\varphi_{w,r}\right)g\left(\varphi_{w,r}\right) \end{array}\;,$$

where $d_{i,w}$, $\varphi_{i,w\;}$, and $\omega_{k,w}$ are the distances, receiving incident angle, and the irradiance angle between the *i*^th^ Tx and the reflective area, respectively. $d_{w,r}$, $\varphi_{w,r}$, and $\omega_{w,r}$ are the distances, receiving incident angle, and irradiance angle between the reflective area and the Rx, respectively. *ρ* is the reflectance factor of the reflecting surfaces and $A_{\mathrm{ref}}$ is the reflectance area. For the NLoS case, a significant error may occur when calculating the distance due to the existence of reflections, as noted in (5). Therefore, a polynomial fitted model is introduced to express the relation between $P_{R,i}\;$and the total distance from *i*^th^ Tx and the Rx [32,33], which is given by:

(6) $$d_{i}\left(P_{R,i}\right)=a_{0}+a_{1}P_{R,i}+a_{2}{\left(P_{R,i}\right)}^{2}+\ldots +a_{g}{\left(P_{R,i}\right)}^{g},$$

where $a_{0\;\cdots }a_{g}$ are the coefficients of the polynomial model for a $g^{\mathrm{th}}$ order polynomial.

### 2.2. Estimation Algorithms

In the case of LLS, $a_{g}$ values are initially estimated based on the fitting process for the given values of $d_{i}$ and $P_{R,i}$. These values are then utilized for the estimation of $d_{i}\;$and substitution in (4) to determine $r_{i}$ for each Tx. Note that, LLS is used to find a coarse estimate of the Rx’s position, which is given by [18]:

(7) $$\hat{X}=\left[\begin{array}{c} {\hat{x}}_{\mathrm{Rx}}\\ {\hat{y}}_{\mathrm{Rx}} \end{array}\right]={\left(A^{T}A\right)}^{-1}A^{T}B,$$

where $\left[{\hat{x}}_{\mathrm{Rx}},{\hat{y}}_{\mathrm{Rx}}\right]$ is the estimated position of the Rx, and *A* and *B* are given as:

(8) $$A=\left[\begin{array}{cc} x_{2}-x_{1} & y_{2}-y_{1}\\ \vdots & \vdots \\ x_{I}-x_{1} & y_{I}-y_{1} \end{array}\right],\;\;B=0.5\times \left[\begin{array}{c} \left(r_{1}^{2}-r_{2}^{2}\right)+\left(x_{2}^{2}+y_{2}^{2}\right)-\left(x_{1}^{2}+y_{1}^{2}\right)\\ \vdots \\ \left(r_{1}^{2}-r_{I}^{2}\right)+\left(x_{I}^{2}+y_{I}^{2}\right)-\left(x_{1}^{2}+y_{1}^{2}\right) \end{array}\right],$$

where *I* is the total number of Txs. However, the LLS estimation solution may not offer a high positioning accuracy [18]. This is especially true for the positions close to the walls and corners, where the signal power levels from the NLoS paths are higher. The NLLS estimation can be utilized as an alternative approach for position estimation, which minimizes the approximation error attained from LLS estimation [25]. The trust region algorithm is employed to solve the unrestricted optimization problem to realize the 3D positioning [34]. The estimated location is found at the minimum of the averaged squared error $\tilde{C}$, which is given by:

(9) $$\tilde{C}=\overset{I}{\underset{i=1}{\sum }}{\left(\sqrt{{\left({\tilde{x}}_{\mathrm{Rx}}-x_{i}\right)}^{2}+{\left({\tilde{y}}_{\mathrm{Rx}}-y_{i}\right)}^{2}}-r_{i}\right)}^{2},$$

where ${\tilde{x}}_{\mathrm{Rx}}$ and ${\tilde{y}}_{\mathrm{Rx}}$ is the estimated position of the Rx. $r_{i}$ is computed from (4) and (6). In this work, we consider NLLS with a polynomial fitted model for the distance as the baseline for performance comparison.

## 3. The Concept of Neural Network

### 3.1. Use of ANN for Regression

**Figure 4:**
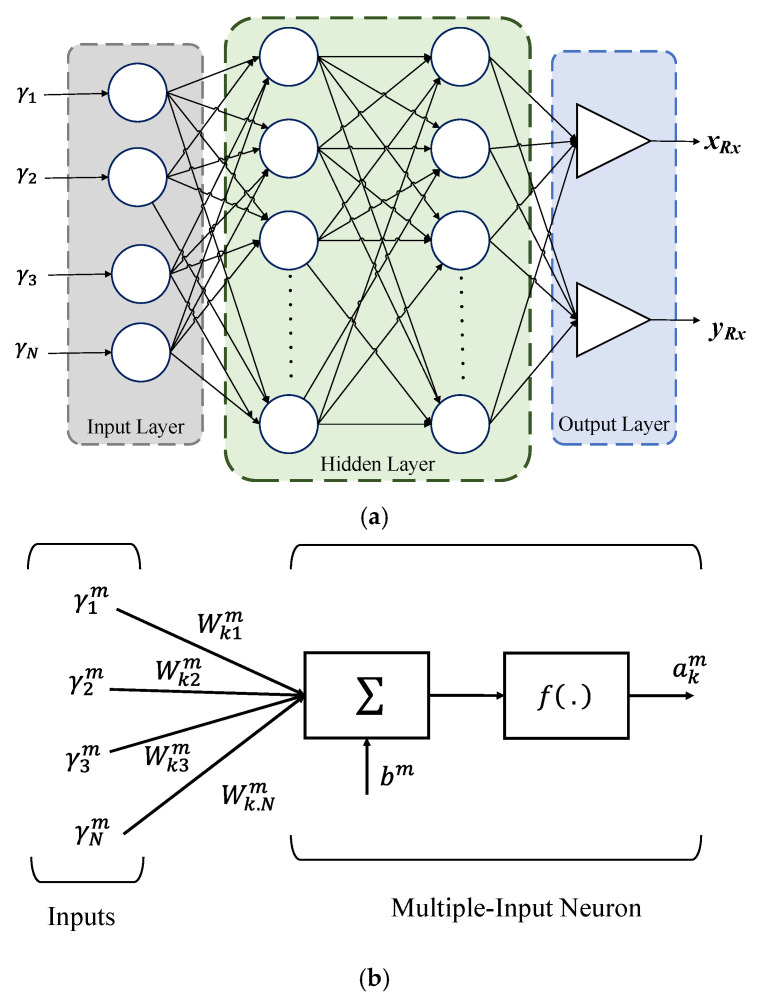
The artificial neural network with: (**a**) a basic structure; and (**b**) a structure of *k*^th^ neuron with *N* inputs in the layer *m*.

Even with the power versus the distance relation for NLoS described in (6), the room morphology (corners, walls, furniture, etc.) changes a great deal, thus making it difficult to infer an approximate model, which is applicable for every scenario. As a result, using ANN is advantageous since it is trained using $P_{R,i}$ from each Tx and the transmission distance. The regression analysis is useful to model the relationship between a dependent variable and one or more independent variables (i.e., the input values in the model). One of the possible solutions for any type of regression problem is the ANN. The ANN is inspired by the process of the human brain, and therefore is composed of neurons that work in parallel. Each neuron is capable of performing a simple mathematical operation individually [35]. Collectively, the neurons can evaluate complex problems, emulating most of the functions and providing precise solutions. The ANN is an interconnected network of processing elements (neurons) and it includes two different phases: (i) the training phase-where the ANN estimates an input-output map based on the training data set. During this training phase, the neuron weights are continuously adapted to minimize the error between the estimated output and the training data vectors. The process terminates when the required performance is achieved, or the complete training set is used; and (ii) the operation phase-where the ANN is employed to perform estimates based on the input data alone. The ANN structure consists of at least three layers; a single input layer consisting of γ*_N_*, one or several hidden layers (HL), and a single output layer (see Figure 4a). These layers are linked together based on a collection of connected units or nodes, called the artificial neurons. The importance of these neurons is defined based on their weights and the learning process.

The weight$\;W_{kn}^{m}$ has the capability to acquire and store experimental knowledge, where *k*, *n*, and *m* represent the number of neurons, inputs, and layers, respectively. These are also known as the synaptic weights as their principle is like the synapses present in biological brains. It relates the *n*^th^ input to the *k*^th^ neuron. Note, the number of neurons in the hidden layer controls the weights and the bias in the network. Each neuron can be biased with a value *b^m^* as depicted in Figure 4b. For HLs, a sigmoid transfer function is used as an activation function that applies thresholding to the input data and produces outputs as a continuous value between zero and one, while the output layer employs a linear transfer function. The performance of an ANN algorithm is measured by the mean square error, which can be expressed as a function of *F*($p_{k}^{m}$) as:

(10) $$F\left(p_{k}^{m}\right)=e_{k}^{m}=∥t_{k}^{m}-a_{k}^{m}{∥}_{{}_{2}},$$

where $p_{k}$ is the vector containing all of the network weights and biases for the *k*^th^ neuron (i.e., $p_{k}$ = $\left[W_{k},\;b_{k}\right]$), and $a_{k}^{m}$ is the network output of the *k*^th^ neuron for the *m*^th^ layer and $t_{k}^{m}$ is the target output of the *k*^th^ neuron for the *m*^th^ layer. The weights and the bias are updated by the backpropagation method [35] as:

(11) $$W_{k,n+1}^{m}=W_{k,n}^{m}-Gs^{m}{\left(a_{k}^{m-1}\right)}^{T}$$

(12) $$b_{k,n+1}^{m}=b_{k,n}^{m}-Gs^{m},$$

where G is the learning rate, m=0,1,…,M−1, *M* is the number of layers in the network, and (.)^T^ is the transpose. $b_{k,n}^{m}$ is the bias vector. $\gamma_{kn}^{m}$ is the input vector, n=0,1,…,N, and *N* is the total number of inputs in the network. $s^{m}$ is the sensitivity matrix, which is evaluated from the least mean square error function, $\hat{F}\left(p_{k}^{m}\right)$ for various values of j, wherein *j* is defined in the matrix form as $\gamma_{k}W_{k}+b_{k}.$

The ANN structure in the proposed study is composed of four layers: an input layer; two HLs; and an output layer. Each layer has a different number of neurons, with the input and output layers having four and two neurons, respectively. The estimated *x* and *y* position coordinates are represented by the output neurons. The estimated distances from each Tx are applied to the input layer with the help of (6).

In this work, we have investigated the number of HLs and have determined that a simple ANN with only one hidden layer would not provide the desired results, i.e., high positioning errors. Using two hidden layers provided a more effective framework for achieving improved performance. Therefore, based on our preliminary research, we limited the number of hidden layers to two. The neurons in the HLs are activated using a Sigmoid transfer function, which thresholds the input data and outputs a continuous value between zero and one. A linear transfer function is used in the output layer. All notations utilized in the paper are indicated in Table 1.

Following that, we have adopted a few well-known training algorithms and used them to analyze the $\varepsilon_{p}$ of the proposed system. For this investigation, we have used the default values of Matlab’s fitnet tool to fix the parameters such as the learning rate. Note that, other parameters such as the number of neurons in HLs or the activation functions could also be optimized based on the topology of the HLs. Since Sigmoid and linear activation functions have been shown to perform well in regression tasks [36], therefore, they are used in the hidden and output layers, respectively. Having selected Bayesian regularization as the optimal learning algorithm, we then optimized the learning phase using the number of epochs and size of the training set.

### 3.2. ANN Training Methods

The network records the trained information in $W_{kn}^{m}$ and $b^{m}$. Supervised learning algorithms are adopted in this work as explained in the following subsections.

#### 3.2.1. Levenberg–Marquardt Algorithm

The Levenberg–Marquardt (LM) algorithm is employed to solve the NLLS problems. By leveraging the most used optimization algorithms (i.e., Gauss–Newton algorithm, and the steepest descent algorithm), the LM algorithm can avoid some problems, such as over-parameterization, local minima, and non-existence of the inverse matrix [37]. Moreover, it inherits the speed advantage of Gauss–Newton algorithm and the stability of the steepest descent algorithm. The updated rule of weights and biases, i.e., $p_{k}$ is given by:

(13) $$p_{k+1}=p_{k}-{\left[\;J_{k}^{T}\;J_{k}+\mu_{k}I\right]}^{-1}-J_{k}e_{k},$$

where $\;J_{k}$ is Jacobian matrix of the function, $F\left(p_{k}\right)$, and $\mu_{k}\geq 0$ is a scalar, and *I* is the identity matrix. 

#### 3.2.2. Bayesian Regularization Algorithm

Bayesian regularization (BR) is an algorithm that updates the values of weight and bias in accordance with LM optimization. In this algorithm, firstly, a linear combination of the squared errors and the weights are minimized and then, the linear combination is modified with the aim of obtaining a network with good generalization qualities [35]. In BR, the mean squared error function can be defined as:

(14) $$F\left(p_{k}\right)=\beta E_{D}+\alpha E_{W},$$

where $E_{D}\;$is the squared error, $E_{W}$ is the sum of squared weights, which penalizes large weights in reaching a better generalization and smoother mapping, α, and β are the regularization parameters (or objective functions), which are given as:

(15) $$\;\alpha =\frac{\gamma_{e}}{2E_{W}\left(p_{k}\right)},\;\beta =\frac{N_{wb}-\gamma_{e}}{2E_{D}\left(p_{k}\right).},$$

where $\gamma_{e}=N-2\alpha \mathrm{tr}\left(H^{-1}\right)\;$ is called the effective number of parameters, $H=\nabla^{2}F\left(p_{k}\right)\;$ is Hessian matrix, $N_{wb}$ is the total number of parameters (weights and biases) of the network, $\mathrm{tr}\left(H^{-1}\right)$ is the trace of the inverse of Hessian matrix. Note, the 2nd term in (15) is known as the weight decay, and therefore small values of *W* would reduce the overfitting of the model.

#### 3.2.3. Scaled Conjugate Gradient Algorithm

Most of the conjugate gradient algorithms use a line search for each iteration, thus making them computationally complex. Therefore, to address this we have adopted the scaled conjugate gradient (SCG) algorithm developed by Moller [38]. SCG is based on conjugate directions without performing a line search, with reduced computational complexity. The SCG algorithm, which is a scaled conjugate gradient method for updating the weight and bias values, is robust and does not depend on the user-defined parameters, given that the step size is a function of quadratic approximation of the error [38]. Different approaches are used for estimating the step size, which is given by:

(16) $$\xi_{k}=\frac{\mu_{k}}{\delta_{k}}=\frac{-{\bar{p}}_{k}^{T}E_{qw}^{\prime }\left(p_{k}\right)}{{\bar{p}}_{k}^{T}{\bar{s}}_{k}+\lambda_{k}{\left|{\bar{p}}_{k}\right|}^{2}},\;$$

where $E_{qw}^{\prime }\left(p_{k}\right)$ is the quadratic approximation of the error function, $F\left(p_{k}\right)$. ${\bar{p}}_{1},\;{\bar{p}}_{2},\ldots .{\bar{p}}_{k}$ are the set of non-zero weight vectors, and ${\bar{s}}_{k}$ is the second-order information. $\lambda_{k}$ is the scaler to be updated such that:

(17) $$\lambda_{k}=2\left(\lambda_{k}-\frac{\delta_{k}}{{\left|{\bar{p}}_{k}\right|}^{2}}\right).\;$$

If $\Delta_{k}>0.75$, then $\lambda_{k}\;$=$\;\lambda_{k}/4$, and if $\Delta_{k}<0.25$ then $\lambda_{k}$=$\lambda_{k}+\delta_{k}\left(1-\Delta_{k}\right)/{\left|{\bar{p}}_{k}\right|}^{2}$. $\Delta_{k}$ is a comparison parameter given by:

(18) $$\Delta_{k}=\frac{2\delta_{k}\left[F\left(p_{k}\right)-F\left(p_{k}+\xi_{k}{\bar{p}}_{k}\right)\right]}{\mu_{k}^{2}}.$$

## 4. Results and Discussion

The proposed system adopted in Section 2 is implemented in the simulation environment using MATLAB. Both NLLS and different ANN algorithms are applied to the proposed VLP system, and the performance of all algorithms is compared. The ANN structure is composed of four layers, which include an input layer, two HLs, and an output layer. The number of neurons in each layer is variable, with four and two neurons in the input and output layers, respectively. The latter represents the estimated *x* and *y* position coordinates. Using (6), the calculated distances from each Tx are fed to the input layer. A sigmoid transfer function is used as the activation function for the neurons in the HLs, which thresholds the input data and provides the output as a continuous value between zero and one. The output layer employs a linear transfer function.

Besides, the proposed positioning process includes: (i) the total received power computed at the Rx; (ii) the polynomial regression model used to determine the power distance relation, and the distance from each Tx to the Rx; (iii) the computed distance is used as the input to the ANN algorithm for training purpose; and (iv) the position is estimated as the output of the ANN algorithm. Furthermore, for the real implementation, the use of these algorithms would imply two phases: the training phase, where previously collected data will be used for training the ANN; and the stand-alone phase, where the trained ANN with fixed weights will be used in the hardware for position estimation.

**Figure 5:**
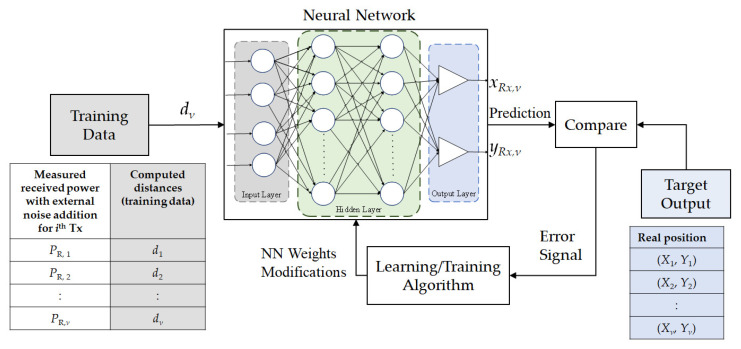
The layout of the neural network used with four layers.

Figure 5 illustrates the overview of the neural network used in the proposed system. The training data consist of different samples, *ν* of inputs and outputs, where *ν* is the total number of samples. The distances are considered as inputs, which are computed by (6). The real position of the Rx, (*X*, *Y*) is considered as the output for the training data. The training data is fed to the neural network for training and the prediction output, (*x_Rx_*, *y_Rx_*) is obtained as estimated positions. These estimated positions are further compared with real positions and the error is again sent to the training algorithm for the modification of weights. This process continues until the network is fully trained.

In this study, two datasets are considered for training, testing, and validation of the ANN as detailed in Table 2. These datasets are composed of the received power information for a given grid of Rxs with different noise power levels (according to the SNR). Note: (i) the data samples are randomly scrambled; and (ii) different datasets are used to avoid biasing of the training process, that is, ANN optimization is conducted using a single dataset, while for the validation and testing, another dataset is adopted. Therefore, 80% of dataset A is used for training, while 20% of dataset B is used for validation and testing. Data scrambling is used to feed the data randomly to the inputs of the neural network for training the network. We consider a grid (1 cm resolution) of 3600 Rx’s positions on the receiving plane, which is divided into two regions, i.e., the inner region where the received power is more uniform and includes the area of the receiving plane within the Txs (LEDs), and the outer region representing the remaining area near the walls and corners as depicted in Figure 1. All the other key system parameters are given in Table 3.

### 4.1. VLP Error Performance

Generally, RSS-based positioning algorithms are susceptible to the ambient induced shot noise, thus leading to increased $\varepsilon_{p}$. In this work, we consider the impact of noise, which is modelled as Gaussian with *N* (0,$\;\sigma^{2}$), on the performance of VLP. A total of 1000 iterations are performed in this simulation to gain some statistical significance. The performance evaluation of the VLP system is provided in terms of the Quantile function Q, which is a valid performance metric to show the level of accuracy. The measurement of the confidence interval of $\varepsilon_{p}$ is carried out through the performance metrics of the Q, which is given by [26]:

(19) $$Q\left(\eta \right)={\mathrm{CDF}}^{-1}\left(\eta \right),$$

where CDF represents the cumulative distribution function of $\varepsilon_{p}$, and η is the percentage of the confidence interval.

**Figure 6:**
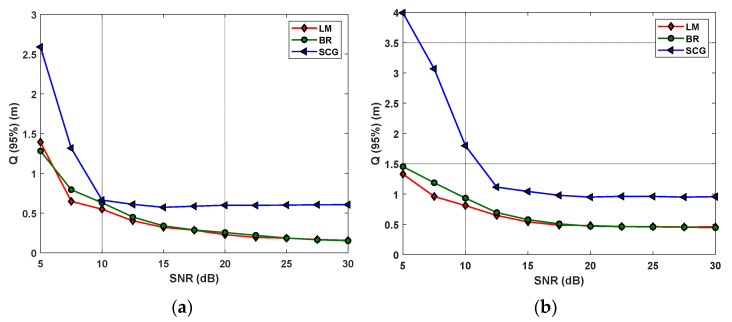
The measured 95% quantile function for different ANN algorithms for: (**a**) the inner; and (**b**) the outer regions.

Figure 6 shows the measured $Q\left(95\%\right)$ as a function of the SNR for different ANN algorithms in both inner and outer regions. It is observed that, LM and BR outperform SCG in both regions. For instance, in the inner region at the SNR of 10 dB, $\varepsilon_{p-\min}$ are 54, 62, and 66 cm for LM, BR, and SCG, respectively, which increases to 80, 95, and 170 cm, respectively, for the outer region. Note, the SNR thresholds for the inner and outer regions are 10 and 15 dB, respectively, where beyond these values, the positioning errors remain almost constant at the lowest levels. Note that, we have considered the average SNR values in the analysis. The decreasing trend in the $\varepsilon_{p}$ is justified by the increase in SNR. For high values of SNR, the effect of noise on the estimated position is reduced. On the contrary, for the small values of SNR, the randomness of the input data leads to overfitting, thus making the estimated error larger. To improve the proposed VLP system, we further investigate the impact of ANN algorithms, the number of neurons in the HLs, and the epochs in the following sections.

### 4.2. Selection of the Training Algorithm and Number of Neurons in the HL

The number of neurons in the HL and different training methods are investigated in this subsection to determine the optimum algorithm based on $\varepsilon_{p-\min}$. The accuracy of the inner region is higher than the outer region due to more reflections being considered in the corners of the room. Therefore, we have only considered the inner region for the selection of the number of neurons in both HLs. As depicted in Figure 6, both LM and BR have lower $\varepsilon_{p}$ compared with SCG, and therefore, are considered for further analysis. Next, we investigate a different number of neurons in the HL and the training for an ideal scenario (i.e., no noise).

**Figure 7:**
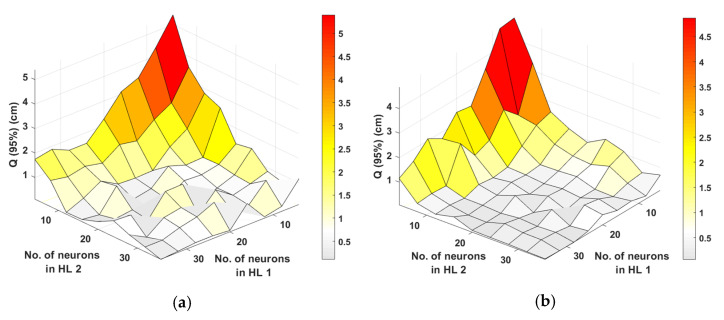
The $\varepsilon_{p}$ for the inner region for different training methods of ANN: (**a**) LM, and (**b**) BR.

Figure 7 shows the surface plots for Q of 95% for the different number of neurons for LM and BR. As depicted in Figure 7, $\varepsilon_{p-\min}$ are 0.11 and 0.06 cm for: (i) LM with 36 neurons each in the HLs of 1 and 2; and (ii) BR with 32 and 28 neurons in HLs 1 and 2, respectively. Based on $\varepsilon_{p-\min}$ the number of neurons in the HL is selected for LM and BR as detailed in Table 4. Note, the training performance is compared for 1000 epochs between LM and BR with the total computation times of 22 and ~10 min, respectively, which are achieved using CPU Intel I Core I i9-9900K CPU @ 3.60 GHz, 3600 MHz, 8 Core PC, having 16 Logical Processors and 32 GB RAM. The epochs represent the number of times the ANN algorithm will run over the full training dataset. BR offers a faster training phase, and therefore, is selected for further investigation of the impact of a different number of epochs.

### 4.3. Impact of Epochs and Noise Performance on the VLP System

**Figure 8:**
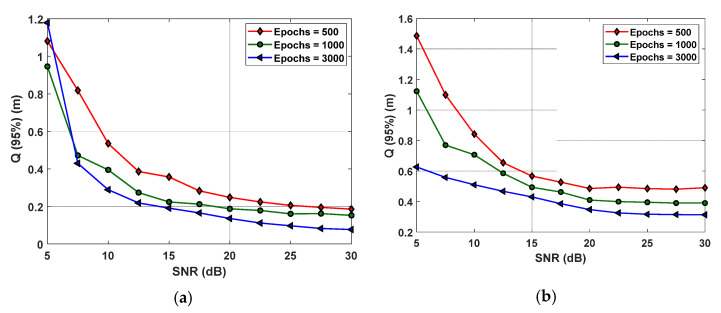
The measured 95% quantile function for a various number of epochs for BR in the: (**a**) inner, and (**b**) outer regions.

Firstly, the effect of epochs in the proposed VLP system is observed, where we investigate different epoch values and their impacts on the error performance. Figure 8 depicts the Q(95%) as a function of SNR for epochs of 500, 1000, and 3000 for inner and outer regions. We can see that, for the inner and outer regions, the epoch of 3000 offers the lowest Q for moderate and high values of SNR, and therefore, it is considered for further analysis with the noise. This shows that BR is strongly affected by the number of training epochs, with a larger number of epochs resulting in more tuned network weights. Note that, the epoch of 3000 does not provide high accuracy for the lower value of SNR due to the fact that the network is not able to generalize as well as moderate to high values of SNR. Generally, the precision of the ANN may improve with the higher number of epochs. However, this neglects the possibility of overfitting, which we observed for a larger number of epochs.

**Figure 9:**
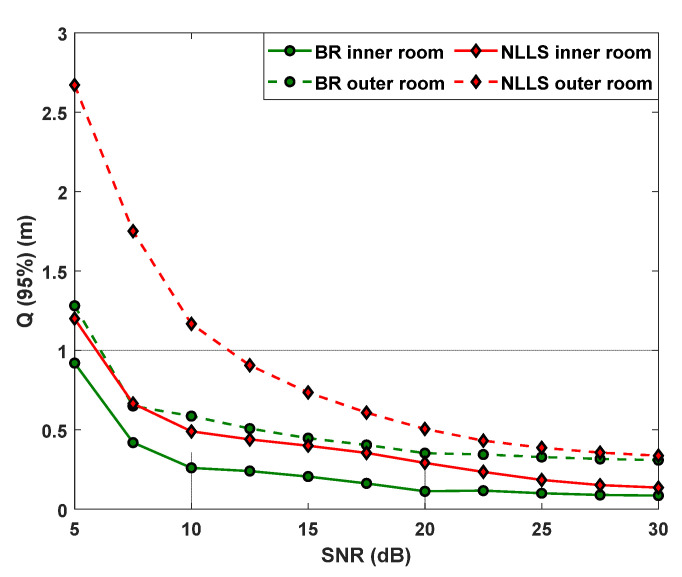
The measured 95% quantile function for NLLS and BR.

Figure 9 depicts the Q(95%) as the function of SNR for the BR-based ANN algorithm and with RSS, as well as for the inner and outer regions and for the epochs of 3000. Results show that NLLS is more prone to the effect of noise and proximity from walls and corners than BR. This can be explained by the ability of the ANN to better estimate the positions near the walls than NLLS and the inherent immunity to the noise. As shown, $\varepsilon_{p}$ is reduced significantly using ANN. For instance, at the SNR of 30 dB and for the inner region $\varepsilon_{p-\min}$ are 8 and 13 cm for BR and NLLS, respectively. Moreover, in the inner region, the accuracy improvement values of 46, 58, and 38% are observed for the SNR values of 10, 20, and 30 dB, respectively. While in the case of the outer region, the accuracy improvements of 50, 30, and 9% are observed for the SNR values of 10, 20, and 30 dB, respectively. Therefore, the BR outperforms the traditional NLLS for the SNR range of 5–30 dB.

**Figure 10:**
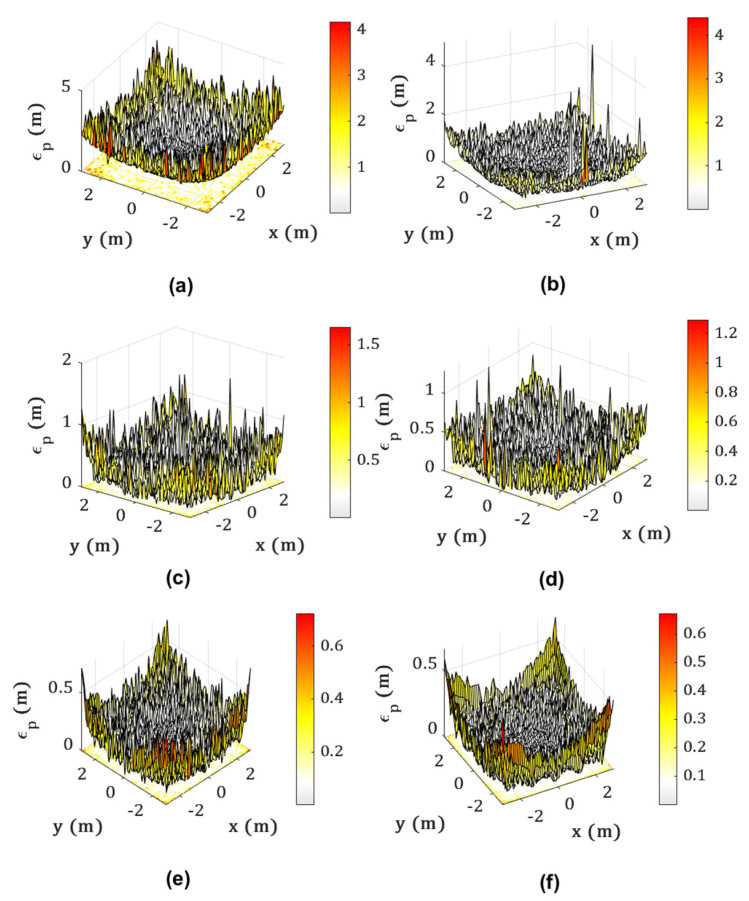
Different error distribution plots using BR algorithm for SNR value: (**a**) 5 dB; (**b**) 10 dB; (**c**) 15 dB; (**d**) 20 dB; (**e**) 25 dB; and (**f**) 30 dB.

Figure 10 depicts the error distribution plots using Bayesian regularization algorithm for different ranges of SNR. It can be observed that the positioning error $\varepsilon_{p}$ decreases by increasing the SNR values. Therefore, we can clearly see the impact of noise in these error plots. The main observations are detailed in Table 5.

### 4.4. Impact of Different Training Dataset Sizes on the VLP System

Furthermore, we analyze the impact of different training dataset sizes denoted by *I_n_* on the Q. For this, we have considered two training scenarios: the random selection (RS), and the uniform selection (US). In the former, the original dataset A is down-sampled from the original 18,000 samples to 9000, 4500, 2250, and 1125 datasets. While in the latter, the grid size is down-sampled from the original 60 × 60 samples to the aforementioned sizes. By doing so, we aim to show if the system performance depends on the selection of training dataset samples. Here, we have only generated results by considering only the data from the inner region.

**Figure 11:**
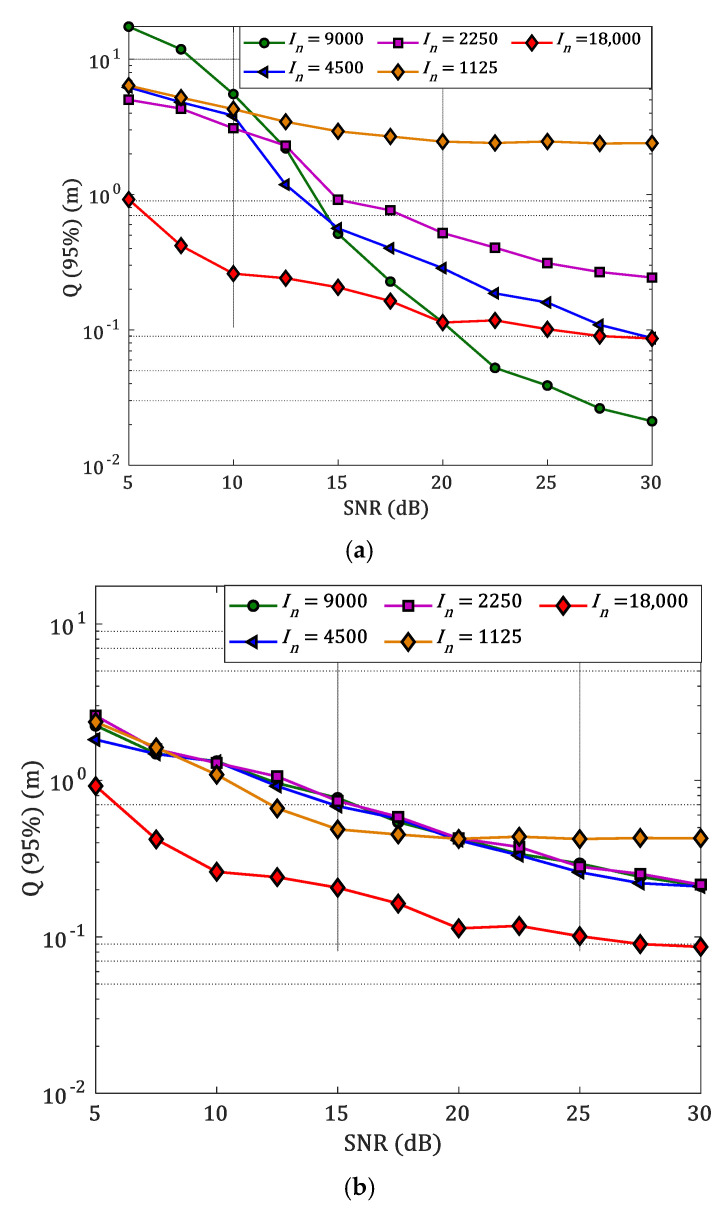
The measured 95% quantile function for a different number of samples in the input with: (**a**) RS; and (**b**) US.

Figure 11 shows the error performance versus the SNR for a range of *I_n_* and for both RS and US scenarios. For the RS scenario, the $\varepsilon_{p-\min}$ values are 2, 11, and 44 cm for the SNR values of 30, 20, and 10 dB, respectively, with a lower *I_n_* of 9000 compared to 15, 22, and 44 cm for the US scenario with a higher *I_n_* of 18,000. Results show that, the US scenario conducts to larger errors, and this is a result of us sampling the grid resolution. This may conduct to overfitting problems. With the RS scenario, the accuracy improves for high SNR values showing that there is an optimum size for the training dataset. This can be attributed to the fact that the original grid resolution is fixed, leading to less probability of overfitting. Therefore, considering the original dataset provides improved results, the proper selection of the training dataset sizes is also essential to properly design the system.

## 5. Conclusions

An indoor VLP system using an artificial neural network for positioning estimation in the presence of both line-of-sight and non-line-of-sight multipath signals was analyzed. In order to implement a realistic scenario, we studied the influence of noise in the proposed system. Three different ANN algorithms of Levenberg–Marquardt, Bayesian regularization, and scaled conjugate gradient algorithms were explored for minimizing the positioning error. The optimization of ANN was conducted based on the number of neurons in the hidden layers and the number of training epochs. We showed that the ANN with Bayesian regularization outperforms the traditional RSS technique using NLLS for the SNR range of 5–30 dB. We also observed an improvement in the positioning accuracy for the inner region by 43, 55, and 50% compared to 57, 32, and 6% in the outer region for the SNR of 10, 20, and 30 dB, respectively. We further studied the impact of different training dataset sizes for training the neural network. It is concluded that, ANN is an efficient method that allows us to achieve a minimum positioning error of 2 cm for 30 dB of SNR with a random selection of training dataset sizes. Finally, we observed that the positioning error is low even for a lower range of SNR, i.e., positioning error values of 2, 11, and 44 cm for the SNR of 30, 20, and 10 dB, respectively. In our future work, we will be developing an experimental test-bed for verification of the simulated results.

## Figures and Tables

**Table 1:** List of notations used in this paper.

Notation	Definition
$\varepsilon_{p}$	Positioning error
*h_t_*	Height of the Tx
*h_r_*	Height of the Rx
*m*	Lambertian mode
$P_{R,i}$	Total received power from the *i*^th^ Tx
$P_{LoS,i}$	Received power from the *i*^th^ Tx due to the path loss
$P_{NLoS,i}$	Received power from *i*^th^ Tx due to NLoS path
$n_{G}$	Additive white Gaussian noise
$d_{i}$	Distance between the *i*^th^ Tx and the Rx
$\omega_{i}$	The irradiance angle from the *i*^th^ Tx to the Rx
*φ*	Incident angle
R	Photodiode responsivity
$P_{t,i}$	Transmitted power from the *i*^th^ Tx
*T_s_* (φ)	Transmittance function
*g* (φ)	Concentrator gain of the Rx
$A_{r}$	Area of the photodetector
$r_{i}$	The horizontal distance from the *i*^th^ Tx to the Rx
*h*	The difference in height between the Tx and Rx, i.e., (*h_t_*− *h_r_*)
$d_{i,w}$ $,\;\varphi_{i,w\;}$ $,\;\omega_{k,w}$	The distances, receiving incident angle, and the irradiance angle between the *i*^th^ Tx and the reflective area, respectively
$d_{w,r}$ $,\;\varphi_{w,r}$ $,\;\omega_{w,r}$	The distances, receiving incident angle, and the irradiance angle between the reflective area and the Rx, respectively
*ρ*	The reflectance factor depending on the material of the reflective surface
$A_{\mathrm{ref}}$	Reflectance area
$a_{0\;\cdots }a_{g}$	Coefficients of the polynomial model for the $g^{\mathrm{th}}$ order polynomial
$\left[{\hat{x}}_{\mathrm{Rx}},{\hat{y}}_{\mathrm{Rx}}\right]$	The estimated position of the Rx
$\tilde{C}$	Averaged squared error
${\tilde{x}}_{\mathrm{Rx}}$ $,\;{\tilde{y}}_{\mathrm{Rx}}$	The estimated position of the Rx.
$W_{kn}^{m}$	Weight
$p_{k}$	The vector containing all the network weights and biases for the $k^{\mathrm{th}}$ neuron i.e., $p_{k}=[W_{k},\;b_{k}]$
$a_{k}$	The network output for the *k*^th^ neuron
$t_{k}$	The target output of the network for the *k*^th^ neuron
G	Learning rate
M	Maximum number of layers
b	Bias vector
*m*	Number of layers
*k*	Number of neurons
γ	Input vector,
*N*	Total number of inputs
n	Number of inputs
$e_{k}$	Error matrix
s	Sensitivity matrix
$\hat{F}(p_{k}^{m})$	Least mean square error function
*F* $\left(p_{k}^{m}\right)$	Mean square error
$J_{k}$	Jacobian matrix
$\mu_{k}$	A scalar
*I*	Identity matrix
$E_{D}$	Squared error
$E_{W}$	Sum of squared weights
α, β	Regularization parameters
$\gamma_{e}$	Effective number of parameters
H	Hessian matrix
$N_{wb}$	Total number of parameters (weights and biases) of the network
$\mathrm{tr}(H^{-1})$	The trace of the inverse of Hessian matrix
$E_{qw}^{\prime }\left(p_{k}\right)$	Quadratic approximation of the error function, $F\left(p_{k}\right)$
${\bar{p}}_{1},\;{\bar{p}}_{2},\ldots .{\bar{p}}_{k}$	The set of non-zero weight vectors
${\bar{s}}_{k}$	Second-order information
$\lambda_{k}$	A Scalar
$\Delta_{k}$	Comparison parameter
η	Percentage of the confidence interval
Q	Quantile function
$\varepsilon_{p-\min}$	Minimum positioning error
$\xi_{k}$	Step size

**Table 2:** Total dataset samples considered for the proposed ANN.

	Dataset A	Dataset B
Grid size	60 × 60	100 × 100
Total number of sample	18,000	50,000

**Table 3:** The key system parameters.

Parameter	Value
Room size	6 × 6 × 3 m^3^
Locations of the Txs	
$(x_{1}$, $y_{1}$, $z_{1})$,	(−1.7, −1.7, 3),
$(x_{2}$, $y_{2}$, $z_{2})$,	(−1.7, 1.7, 3),
$(x_{3}$, $y_{3}$, $z_{3})$,	(1.7, −1.7, 3),
$(x_{4}$, $y_{4}$, $z_{4}$)	(1.7, 1.7, 3)
Area of PD	10^−4^ m^2^
Half-power angle (HPA)	70°
Responsivity of PD	0.5 A/W
Field of view (FOV)	75°
Transmitted power	1 W
Reflection coefficient	0.7
Activation function	Sigmoid, linear
Number of neurons in the input layer	4
Number of neurons in the hidden layer	2–36
Number of neurons in the output layer	2
Number of hidden layers	2
Percentage of train to test	0.8

**Table 4:** Comparison of $\varepsilon_{p-\min}$ for different training algorithms.

Algorithms	$\varepsilon_{p}\;(\mathrm{cm})$	Neurons in HL 1	Neurons in HL 2
LM	0.11	36	36
BR	0.06	32	28

**Table 5:** Final observations of the comparison of BR and traditional RSS with NLLS algorithms.

	BR	RSS with NLLS
Max. *P_R_* (µW)	6.7 × 10^4^	6.7 × 10^4^
Min. *P_R_* (µW)	3.6 × 10^4^	3.6 × 10^4^
Max. $\varepsilon_{p}$ at 20 dB (m)	0.89	1.29
Min. $\varepsilon_{p}$ at 20 dB (m)	16 × 10^−4^	18 × 10^−4^
Max. $\varepsilon_{p}$ at 25 dB (m)	0.71	0.72
Min. $\varepsilon_{p}$ at 25 dB (m)	6.1 × 10^−4^	15 × 10^−4^
Max. $\varepsilon_{p}$ at 30 dB (m)	0.54	0.67
Min. $\varepsilon_{p}$ at 30 dB (m)	5.4 × 10^−4^	4.6 × 10^−4^

## Abbreviations

**Short Form**	**Description**
ANN	Artificial neural network
AOA	Angle of arrival
BR	Bayesian regularization
FOV	Field of view
HLs	Hidden layers
HPA	Half-power angle
LEDs	Light-emitting diodes
LLS	Linear least square
LM	Levenberg-Marquardt
LoS	Line of sight
NLLS	Nonlinear least square
NLoS	Non-line of sight
OOK	On-off keying
PD	Photodiode
RF	Radio frequency
RSS	Received signal strength
Rx	Receiver
SCG	Scaled conjugate gradient
SNR	Signal to noise ratio
TOA	Time of arrival
Txs	Transmitters
VLC	Visible light communication
VLP	Visible light positioning
